# Additive manufacturing in space research: hybrid mass analyser for laser ablation ionisation mass spectrometry

**DOI:** 10.1039/d4ja00392f

**Published:** 2025-02-05

**Authors:** Andreas Riedo, Peter Keresztes Schmidt, Nikita J. Boeren, Salome Gruchola, Luca N. Knecht, Marek Tulej, Peter Wurz

**Affiliations:** a Space Research and Planetary Sciences, Physics Institute, University of Bern 3012 Bern Switzerland andreas.riedo@unibe.ch; b NCCR PlanetS, University of Bern 3012 Bern Switzerland

## Abstract

Additive manufacturing has found its way into many industrial and academic areas. In this contribution, we present an additively manufactured reflectron, integrated in a space-prototype mass analyser used in laser ablation ionisation mass spectrometry. Fused deposition modelling technology was applied to produce the reflectron's ion optical system. For the insulating parts, polylactic acid filament was used as printing material, while the conductive ion optical parts were printed using polylactic acid impregnated with carbon. Measurements were conducted on a stainless steel sample (AISI 316 L, 1.4435) and NIST SRM 661 sample to validate the performance of the reflectron. We found that this system performed nominally in terms of mass resolution and detection sensitivity. This demonstrates the suitability of 3D printing for rapid prototyping in laboratory environments. The latter is of considerable importance for future space exploration missions, as the methodology allows testing of new designs time efficiently and at reduced costs.

## Abstract 2

Additive manufacturing of ion optical components for rapid prototyping in mass spectrometry. 3D printed reflectron shows similar performance to conventionally manufactured system.

## Graphical abstract

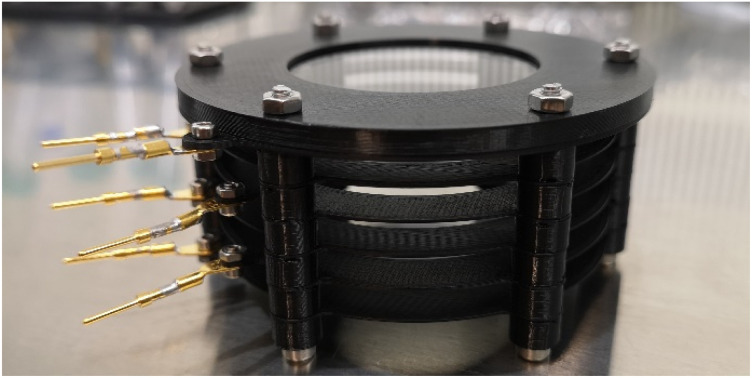

## Introduction

With the availability of affordable 3D printers offering excellent print quality, additive manufacturing is increasingly being used in industrial and academic activities.^1–4^ In recent years, the technology has also been applied in analytical chemistry, *e.g.*, supporting daily laboratory work (*e.g.*, tools for sample preparation or mechanical structures used in measurement setups) and is now increasingly utilised in mass spectrometry and spectroscopy. To give just a few examples, additive manufacturing has been successfully applied to separation technologies,^5^ ion guides for Electro Spray Ionisation (ESI) sources to improve ion transport to the mass analyser,^6,7^ microfluid^8,9^ and microreactor^10^ systems, ion sources and Thin Layer Chromatography (TLC) chips,^9^ an electron impact gas ioniser for compact mass spectrometry,^11^ ceramic cylindrical ion trap mass analyser chips,^12^ and the backbone of a ceramic quadrupole mass spectrometer.^13^ Additive manufacturing has also been used to print specific reference materials, such as Pt group element reference materials.^14^ For reviews on additive manufacturing in mass spectrometric applications and technologies in general, and as well as on different additive manufacturing technologies, we refer readers to the listed publications.^1,2,4,9^

Typically, a space-prototype system used in a laboratory environment requires significant technical adaptation before it can be deployed on a space exploration mission. For example, the high mechanical loads during rocket launch would damage such a system, which must be avoided at all costs. These adaptations can include changes to the spacings between or geometries of the electrodes in the mass analyser's ion optical system, which can affect the instrument's performance. Until now, adapted designs have been produced conventionally, for example using 5-axis CNC machining, and tested with the existing laboratory hardware to identify any problems with the new design before final production of a flight system. This process is time consuming and dependent on the availability of many resources. To overcome this limitation during design testing, we explored additive manufacturing as a rapid prototyping and risk reducing approach in space research using a design of one of our space prototype mass analysers.

In this contribution, we demonstrate for the first time, to the best of our current knowledge, a fully additively manufactured ion mirror consisting of an entrance window integrated with the remaining and conventionally manufactured space-prototype mass analyser (hereafter referred to as the hybrid mass analyser). The ion-optical design represents the core of our Laser Ablation Ionisation Mass Spectrometric (LIMS) system, which is currently being flight-qualified for its deployment on the lunar surface through NASA's Artemis programme.^15–17^ LIMS measurements using the hybrid mass analyser demonstrate the suitability of additive manufacturing in this area of space science and mass spectrometry. This is highly relevant to our field, as design verification can be carried out more efficiently prior to final flight system production. Moreover, new ion optical designs, which so far have not been tested in the laboratory environment, can be produced cheaper, faster, and more independently, which as a result opens new perspectives in this field.

## Experimental

### Sample material

For the validation of the mass spectrometric performance a stainless steel sample (AISI 316L, 1.4435) and the NIST SRM 661 reference material were used. The NIST SRM 661 sample was fixed with UHV compatible copper tape on the sample holder. Measurements on stainless steel were conducted on the sample holder directly, as it is manufactured of said material. The used stainless steel alloy is a standardised material and minimum and maximum values for element abundances are given (all wt%): C (max. 0.03%), N (max. 0.10%), Si (max 1.0%), P (max. 0.045%), S (max. 0.03%), Cr (17.0–19.0%), Mn (max. 2.0%), Ni (12.5–15.0%), and Mo (2.5–3.0%). The chemical composition of NIST SRM 661 is certified and the respective abundances can be found in the corresponding certificate of analysis.

### LIMS instrument

Laser Ablation Ionisation Mass Spectrometric (LIMS) measurements were conducted using the ORganics Information Gathering INstrument (ORIGIN) instrument setup, which is originally designed for the detection of organic molecules. ORIGIN is described in detail in recent publications.^18,19^ Therefore, only a brief description is given here. The setup consists of a miniature reflectron-type time-of-flight mass analyser and a pulsed nanosecond laser system (*λ* = 532 nm, *τ* = 1.3 ns, laser pulse repetition rate of 100 Hz, laser pulse energy of up to about 40 μJ per pulse) used as laser ablation ion source. The latter is different to the nominal ORIGIN setup where we typically operate a UV (*λ* = 266 nm) nanosecond laser system for molecular studies. The mass analyser integrated in ORIGIN is based on a design developed earlier, for the elemental and isotope analysis of solids.^20,21^ We are currently in the design and manufacturing process of a flight LIMS system consisting of the mass analyser design used in this study. Note, that for the flight system only space proven materials and manufacturing processes will be used. The LIMS instrument shall be deployed through a Commercial Lunar Payload Service (CLPS) mission within NASA's Artemis program for the chemical analysis of lunar regolith at locations near the lunar south pole.^16,17^ In said CLPS mission, we will fly a flight version of the microchip laser system used in this study here. The commercially available microchip laser system used in this study is shown in panel A in Keresztes Schmidt *et al.*, 2022.^15^ In this study here, we integrated the same optical tower as shown in Keresztes Schmidt *et al.*, 2022 (ref. 15) on the vacuum chamber, in which the mass analyser is located. The optical tower consists of a beam attenuator and a single lens focusing system. The laser pulses are guided towards the entrance window of the vacuum chamber, through the ion optical system of the mass analyser, onto the sample surface. A laser focus diameter of about 20 μm is obtained. Once the positively charged species are generated through the ablation and ionisation process, they are accelerated and confined into the interior of the mass analyser and are reflected at the reflectron towards the detector system of ORIGIN.^22^ Positively charged species arrive in time sequence at the detector system, according to their mass-to-charge ratio (TOF measurement principle). A high-speed read-out-electronic system is used to record the signal generated by the detector system, and in-house written software is used for data analysis.^23^

The reflectron investigated in this study here consists of five equidistant electrode rings. On top of the reflectron, an active electrode (referred to as backplane) is integrated containing the entrance window of the mass analyser (not to be confused with the entrance window of the vacuum chamber), through which the laser beam is guided. The reflectron is integrated above the drift tube of the mass analyser. In Riedo *et al.*, 2013 (ref. 21) a typical set of voltages applied to the ion optical system, including the reflectron, is provided.

### Additively manufactured reflectron

For the additive manufacturing process of the reflectron, we applied the Fused Deposition Modelling (FDM) technology. This technology is one of the most common processes for printing of 3D components. For a full description of the process and other printing technologies, we refer to *e.g.*, Agrawaal *et al.*, 2021,^1^ Grajewski *et al.*, 2021.^4^ In short, the printing filament is heated up sufficiently (material dependent temperature) and pressed through a printing head (extruder) towards the printing bed. The structure is subsequently printed layer by layer according to the need of the user (*e.g.*, speed, layer thickness, quality, *etc.*).

**Fig. 1:**
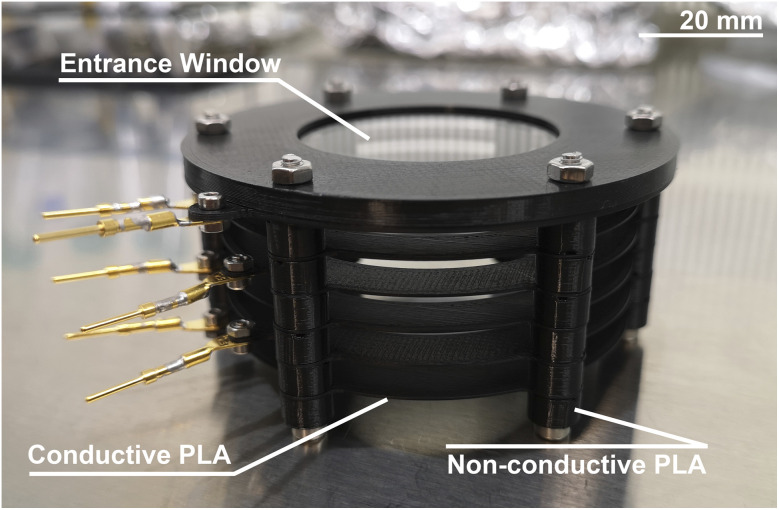
Additively manufactured reflectron of the mass analyser used in LIMS.

For this study, a Sidewinder X1 (Artillery 3D Technology Co., Ltd.) was used for the manufacturing of the reflectron parts. This model allows to print structures with a maximal precision of 50 μm and 100 μm in lateral and vertical directions, respectively. The extruder was equipped with a 0.4 mm nozzle. For the insulating parts (spacers between ion optical elements, insulating tubes, top part of entrance window assembly), normal PLA material (Purefil PLA filament, 1.75 mm core diameter, Fabru GmbH, Switzerland) was used, which showed an electric resistance at the level of MΩ (simple 2-point measurements). Note, screws that go through the insulating parts and ion optical components are used for the integration of the reflectron; they are insulated with the insulating tubes. For active ion optical elements (electrode rings, bottom part of entrance window assembly), conductive PLA material (Protopasta Conductive PLA, Protoplant, Inc., Canada) was used. This material is impregnated with carbon and has an electric resistance at the level of few kΩ per 10 cm of filament (2-point measurements). This is sufficiently low for the operation of our ion optical elements, as they are floated at high voltage and are not power consumers. CAD drawings (STEP files) of each part of the reflectron were imported to the slicer software (PrusaSlicer, Prusa Research a.s., Czech Republic), providing the controlling G-code for the 3D printer. In Table 1, detailed information on the printed parts is listed. For simplicity, normal metallic pins for the electrical contacts are screwed to the ion optical components of the reflectron, to which vacuum compatible cables are subsequently attached. In a more advanced design, even the electrical connections can be realised by the 3D printing process. The additive manufactured reflectron is shown in Fig. 1.

**Table 1:** Additive manufactured parts with their corresponding printing parameters

Part	First layer [mm]	Other layers [mm]	Nozzle temp. [°C]	Bed temp. [°C]
Electrode rings	0.2	0.15	230	50
Insulating spacers	0.25	0.15	200	60
Insulating tube	0.2	0.2	200	60
Bottom side, entrance window	0.25	0.15	230	50
Top side, entrance window	0.2	0.2	200	60

**Fig. 2:**
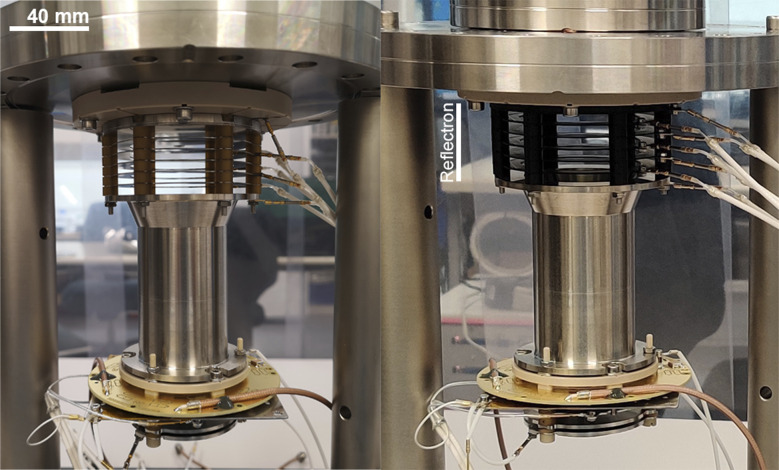
The conventional mass analyser (left) and the hybrid mass analyser with the additively manufactured reflectron in black (right). The mass analyser is integrated into an adapter PEEK plate, which is connected to the top flange of the vacuum chamber.

Similar to CNC machined parts, all manufactured structures were inspected in view of conformity (no PLA whiskers/strings, no overflow of PLA, *etc.*) prior to assembly. After assembly of the additively manufactured reflectron and before integration into the conventional manufactured body of the mass analyser, the reflectron was placed in the ORIGIN vacuum chamber under UHV conditions, to facilitate outgassing. Note, this process is applied as well to conventionally produced parts, allowing a more efficient evacuation of a vacuum chamber prior to instrument operation. After outgassing, which was complete in less than 48 h, the conventional manufactured reflectron was replaced by the additively manufactured one, followed by the integration of the assembled mass analyser into the vacuum chamber of the ORIGIN setup. In Fig. 2, the conventional and hybrid versions of the mass analyser are shown. Reaching the operational vacuum conditions (mid-10^−7^ mbar level) the voltages for the operation of the reflectron were carefully ramped up to the nominal values used in the past before conducting the first LIMS measurements.

### Measurements

Proof-of-concept measurements were conducted on the stainless steel sample. For initial tests, laser pulse energies at the level of about 0.50 μJ (∼120 MW cm^−2^) were tested on the steel substrate and several thousands of TOF spectra were recorded. For these measurements and to avoid possible damages during operation, the LIMS system was not pushed to its limits, *e.g.*, the voltages applied at the detector system were at the lower end.

For the quantification of the measurement performance (mass resolution and detection sensitivity) of the hybrid mass analyser, a pulse energy scan was conducted on NIST SRM 661. Laser pulse energies ranging from about (0.42–0.58) μJ ((103–142) MW cm^−2^, values at the surface) were tested, corresponding to pulse energies just at the ablation threshold of sample material and to conditions where space charge effects impact the measurement capabilities of the mass analyser. The major space charge effects are charging of the surface and coulombic repulsion effects in the plasma plume. For the lower pulse energies applied ((0.42–0.52) μJ) up to 20 000 mass spectra were recorded for each pulse energy. For the two highest pulse energies tested (0.55 μJ and 0.58 μJ) up to 30 000 mass spectra were recorded. For comparison of the performance of the hybrid mass analyser (detection sensitivity and mass resolution), measurements at comparable instrument conditions were conducted on NIST SRM 661 using the conventional mass analyser.

## Results and discussion

**Fig. 3:**
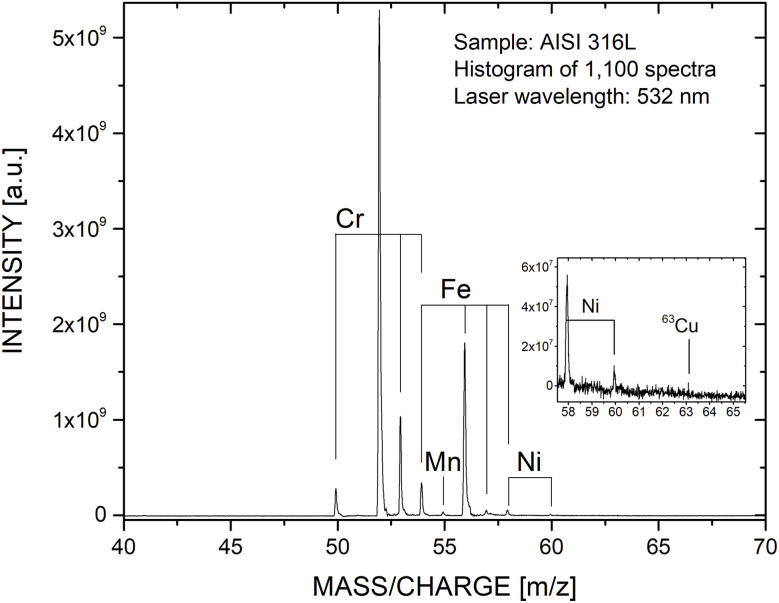
Mass spectrum of stainless steel (AISI 316L, 1.4435) recorded with the mass analyser equipped with the additively manufactured reflectron.

**Fig. 4:**
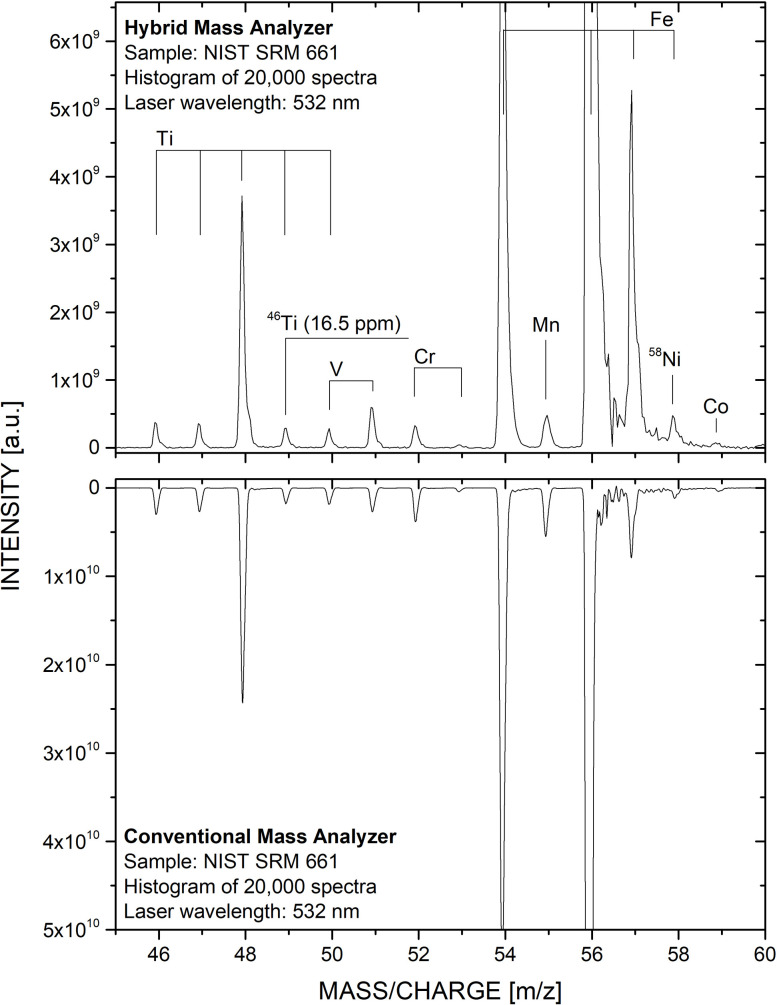
Mass spectrum of NIST SRM 661 recorded with the hybrid mass analyser (upper panel) and conventional mass analyser (lower panel, inverted). Similar measurement performance, such as mass resolution and detection sensitivity, is observed.

In Fig. 3 and 4, the proof-of-concept measurements (Fig. 3) and measurements conducted on NIST SRM 661 (Fig. 4) using the additively manufactured reflectron are shown. Firing the laser on the stainless steel sample directly resulted in well resolved mass spectra at high sensitivity. This was not expected, as typically the ion optical settings need to be slightly adjusted when new hardware is installed at the mass analyser, which was not the case here.

In Fig. 3, a histogrammed mass spectrum of the stainless steel sample is shown. The spectrum consists of 1100 single laser shot mass spectra and was recorded at a laser pulse energy of (0.43 ± 0.02) μJ. In this spectrum, we do see all expected mass peaks (metallic elements), even ^63^Cu just above the noise floor (see inset in the panel). Note, Cu does not belong to the certified list of elements of this stainless steel but abundances of up to 1wt% are accepted according to EN 10088-1. Elements with a high first ionisation potential, such as C or S, are not observed in this proof-of-concept measurement. This observation can be attributed to the applied laser wavelength of *λ* = 532 nm (see *e.g.*, discussion in Riedo *et al.*, 2013 (ref. 24)).

In Fig. 4 two histogrammed (20 000 single laser shot spectra) mass spectrum of NIST SRM 661 are shown. The mass spectrum in the upper panel was recorded using the hybrid mass analyser while the mass spectrum in the lower panel (intensities inverted) was measured using the conventional mass analyser. Both spectra were recorded at comparable laser irradiances at the level of about 110 to 120 MW cm^−2^. Note, at higher laser irradiances peak broadening due to space charge effects is observed. Moreover, higher laser irradiances lead to higher peak intensities, which at some point affect the peak-to-peak baseline separation. Latter effect can be observed for ^56^Fe (Fe being the main constituent of SRM 661), affecting the ^57^Fe peak. By comparing both spectra with each other, a high spectral similarity is apparent. For example, no significant difference in term of mass resolution can be observed; the mass resolution *m*/Δ*m* of ^48^Ti is slightly above 400, which is in line with previous studies (see *e.g.*, Riedo *et al.*, 2013 (ref. 21)). Further, the detection sensitivity is not affected by the additively manufactured reflectron. For example, ^46^Ti, which has a certified atomic fraction of 16.5 ppm, is well above the noise floor in both spectra (*e.g.*, a signal-to-noise ratio of about 100 for the mass spectrum in the upper panel). The quality of the mass spectrum shown here, together with the detection sensitivity quantifiable with NIST SRM 661 (see Fig. 4), is only possible because the additive manufactured reflectron performs as a full-fledged ion optical element perfectly integrating with the ion optics of the remaining mass analyser. Therefore, the printing and integration of the additive manufactured reflectron can be concluded as successful and qualifies the additive manufacturing with the applied FDM technique for mass spectrometric prototyping in this field.

As with conventional CNC machining of ion optical components for high performance mass spectrometric systems, parameters such as surface quality (*e.g.*, planarity and surface roughness), sharp edges, materials used, manufacturable geometries, *etc.* that affect electric fields, need to be investigated in detail in future studies using additive manufacturing. Imperfections in such components could, for example, lead to discharges between ion optical components and thus cause serious damage to mass spectrometric systems. The additive manufactured reflectron presented in this study may not be the most complicated ion optical system, but it is a first and important step towards the validation and application of more sophisticated ion optical parts for high performance mass spectrometric systems.

### Implications for space applications

Once a proposed mass spectrometric system is selected for a space exploration mission, only a laboratory prototype system is typically available. To make this system flight ready, many engineering steps need to be mastered, ranging from material selection, making the system light and robust, to coatings on the structures to make the system inert against the harsh environmental conditions in space. As a consequence, many tests need to be successfully concluded at sub-unit level before the complete system will be manufactured, assembled, and tested. Unfortunately, this requires many production steps as well, which impact the required testing activities. From mass spectrometry point of view, distances and shapes of ion optical components of the laboratory prototype-system need to be adapted to make the mass spectrometer flight ready. To minimise the technical risks associated with the implemented changes on the mass spectrometric unit, additive manufacturing could be integrated in the manufacturing and testing phases prior to final flight hardware production using flight proven materials and machining. In case the design is not working as expected, the part can be adapted and printed without human interaction using additive manufacturing. The part can be then integrated and verified once the setup is ready for follow-up test activities. This allows for rapid iterations in the development process, optimising the available time for the project.

## Conclusions

Space research instrumentation has to survive high mechanical and thermal stresses during their application in space. Consequently, many sub-unit tests must be successfully concluded before integration and testing of a full payload. This also holds for mass spectrometric instrumentation, which measure the chemical composition of *e.g.*, solids on the Martian surface. To make a mass spectrometric system flight-ready starting from a laboratory system design, almost every part of the instrument needs to be adapted, potentially affecting the measurement performance of the system. In this study, the reflectron of a space-prototype LIMS mass analyser design was additively manufactured using FDM technology. Laser ablation studies were conducted on stainless steel and NIST SRM 661 samples. The measurements conducted show that mass spectral quality was not affected by the integration of the additively manufactured reflectron. This represents a true success, as this opens doors for follow-up strategies in mass spectrometry in space science and in general, including rapid prototyping in the laboratory environment and development of ion optical designs that might be too complicated to manufacture using conventional CNC machining.

## Data availability

Data for this article are available at Bern Open Repository and Information System at https://doi.org/10.48620/74876.

## Author contributions

AR was involved in conceptualisation, formal analysis, investigation, methodology, validation, visualization, and writing original draft. PKS was involved in investigation, methodology, and reviewing and editing of the manuscript. NJB, SG, LNK, and MT were involved in reviewing and editing of the manuscript. PW was involved in funding acquisition, project administration, resources, and reviewing and editing of the manuscript.

## Conflicts of interest

There are no conflicts to declare.
